# FitEllipsoid: a fast supervised ellipsoid segmentation plugin

**DOI:** 10.1186/s12859-019-2673-0

**Published:** 2019-03-15

**Authors:** Bastien Kovac, Jérôme Fehrenbach, Ludivine Guillaume, Pierre Weiss

**Affiliations:** 1 ITAV, CNRS, Université de Toulouse, 1 Pl. Pierre Potier, Toulouse, 31106 France; 2 IMT, CNRS, Université de Toulouse, 118, route de Narbonne, Toulouse, 31400 France

**Keywords:** Supervised segmentation, Ellipsoid, Icy plugin

## Abstract

**Background:**

The segmentation of a 3D image is a task that can hardly be automatized in certain situations, notably when the contrast is low and/or the distance between elements is small. The existing supervised methods require a high amount of user input, e.g. delineating the domain in all planar sections.

**Results:**

We present FitEllipsoid, a supervised segmentation code that allows fitting ellipsoids to 3D images with a minimal amount of interactions: the user clicks on a few points on the boundary of the object on 3 orthogonal views. The quantitative geometric results of the segmentation of ellipsoids can be exported as a csv file or as a binary image. The core of the code is based on an original computational approach to fit ellipsoids to point clouds in an affine invariant manner. The plugin is validated by segmenting a large number of 3D nuclei in tumor spheroids, allowing to analyze the distribution of their shapes. User experiments show that large collections of nuclei can be segmented with a high accuracy much faster than with more traditional 2D slice by slice delineation approaches.

**Conclusions:**

We designed a user-friendly software FitEllipsoid allowing to segment hundreds of ellipsoidal shapes in a supervised manner. It may be used directly to analyze biological samples, or to generate segmentation databases necessary to train learning algorithms. The algorithm is distributed as an open-source plugin to be used within the image analysis software Icy. We also provide a Matlab toolbox available with GitHub.

**Electronic supplementary material:**

The online version of this article (10.1186/s12859-019-2673-0) contains supplementary material, which is available to authorized users.

## Background

### Starting observation

Segmenting ellipsoidal structures in 2D or 3D imagescan be used to characterize the shape of organs, tissues, cells, nuclei or other cell organels [1–3], or serve as an initialization for more advanced algorithms such as active contours [4–7].

While fully automatic detection algorithms [8–11] are probably the ideal tool to limit subjectiveness and time of analysis, existing strategies are not sufficient to provide convincing segmentation results when images suffer from strong degradations (e.g. blur, noise, low resolution) or contain densely packed objects. In addition, automatic methods usually require tuning a few parameters, which may be more time consuming than using a simple supervised segmentation algorithm. Finally, the generation of learning databases or gold standards to test and compare existing segmentation algorithms still requires efficient supervised algorithms. Unfortunately, to the best of our knowledge, there currently exists no such freely available tool, which would benefit many different communities.

### Contributions

These few considerations motivated us developing two simple plugins for the Icy image analysis software [12] that are based on a novel computational approach. They are dedicated to fitting ellipses in 2D images or ellipsoids in 3D images. The objectives of this paper are to present the methodology and describe the plugin for 3D ellipsoids.

The core of the algorithm consists in solving the well studied problem of ellipsoid fitting from point clouds. This is a notoriously difficult problem that attracted the attention of researchers from different fields such as computer vision, statistics or numerical analysis, to name a few [13–26]. We propose an original and robust computational algorithm that shares the same spirit as a recent work [26], but significantly outperforms it when the ellipsoids are not centered or anisotropic. An important feature of the proposed algorithm is affine invariance: the point cloud is registered prior to computation, ensuring a robust behavior whatever the shape of the point cloud.

The proposed algorithms are shared not only within the Icy plugin, but also through a set of Matlab codes delivered on a Github repository [27]. To the best of our knowledge, FitEllipsoid is the first open-source toolbox that allows fitting ellipsoids and not more general quadrics (e.g. hyperbolas).

In order to showcase the usefulness of the plugin, we propose to examine the morphology and the distribution of shapes of nuclei in a 3D tumor spheroid. Are they rather elongated, spherical or none of these? Using FitEllipsoid, we obtained the shape of hundreds of nuclei from 3D SPIM images of optically-cleared spheroids.

## Implementation

### Specifications

The main objective of this plugin is to provide users an accurate segmentation of ellipsoidal objects, while satisfying the following constraints:

- permit 3D visualization to allow for visual inspection of the segmentation,
- minimize the time required for user interaction. This is particularly important in biology where hundreds or thousands of objects have to be analyzed routinely,
- export the results as files that other programs can use for further processing,
- deliver a free and open-source software.

### Description

The need for a free software dedicated to biomedical imaging oriented us to the recently developed imaging tool Icy [12]. It is based on VTK (Visualization ToolKit) [28], allowing for nice 3D visualization.

An ellipsoid can be represented in different ways:

- A center (3 parameters), three angles of rotations and the length of each axis (3 parameters).
- A center (3 parameters), the three axes (9 parameters linked through orthogonality relationships) and the length of each axis (3 parameters).
- A center (3 parameters) and a positive symmetric definite matrix (6 coefficients).

Unfortunately, none of these representations can be easily used by a human. For instance, finding the center of the ellipsoid precisely by just looking at the image would result in inaccurate results.

**Fig. 1:**
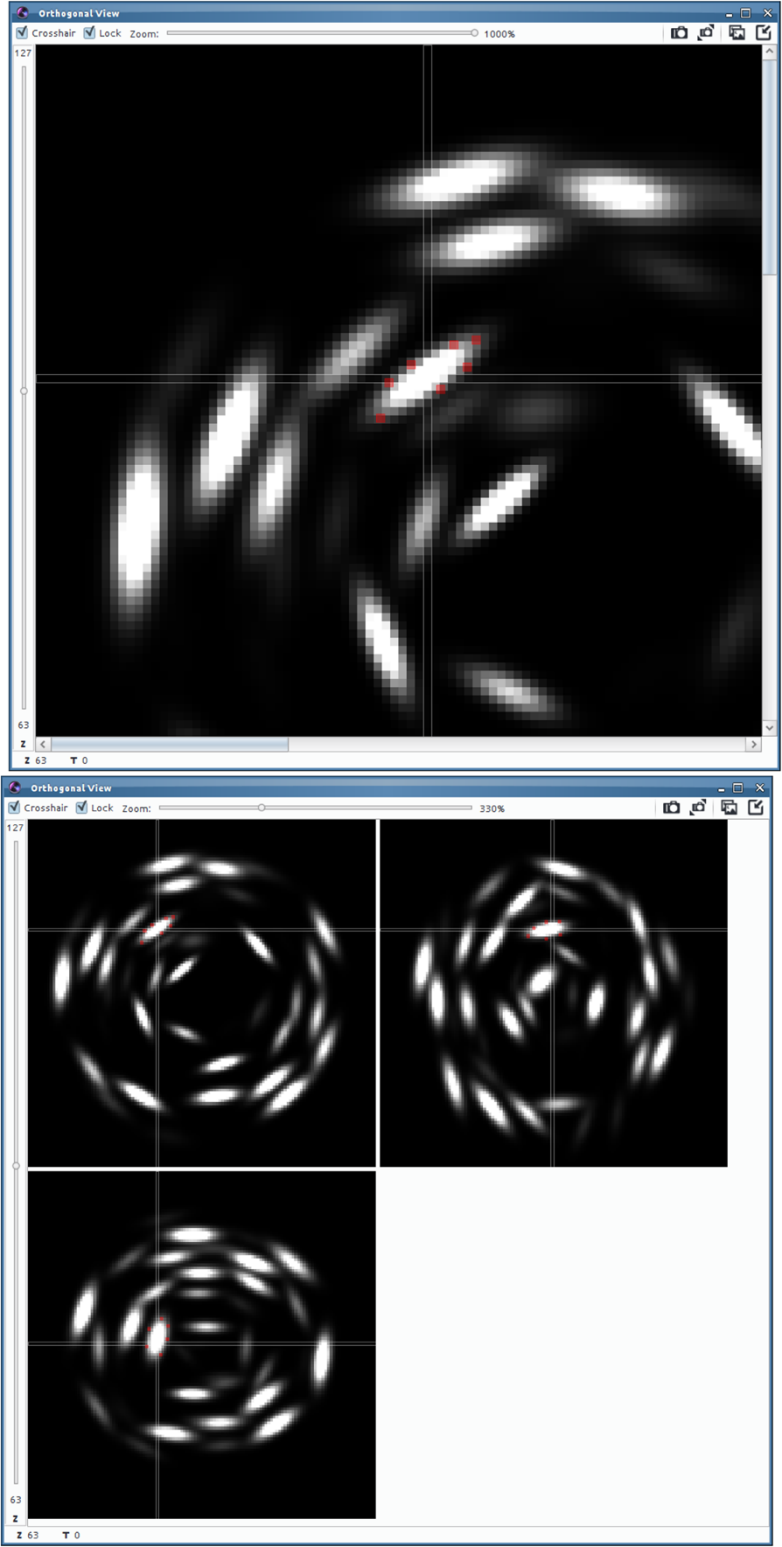
Selecting points in the orthogonal views on a synthetic 3D image. Top: in red the points selected by the user. Bottom: the 3 orthogonal views. By convention the views are (clockwise from top left) in the respective planes (XY, YZ, XZ)

The strategy that is adopted in FitEllipsoid is to ask the user to select a few points in 3D on the object’s boundary and the plugin then creates an ellipsoid that passes through them approximately. In order to select points on the object boundary, we let the user select points on 3 orthogonal 2D views (see Fig. 1).

In theory, it is possible to reconstruct an ellipsoid perfectly when knowing as little as 9 points in generic position lying on its surface (see the Additional file 1 for a detailed discussion). The estimation with just 9 points may be unstable to noise, which cannot be avoided due to imperfect pixel selection by the user. We therefore let the user select as many points on the boundary as desired.

**Fig. 2:**
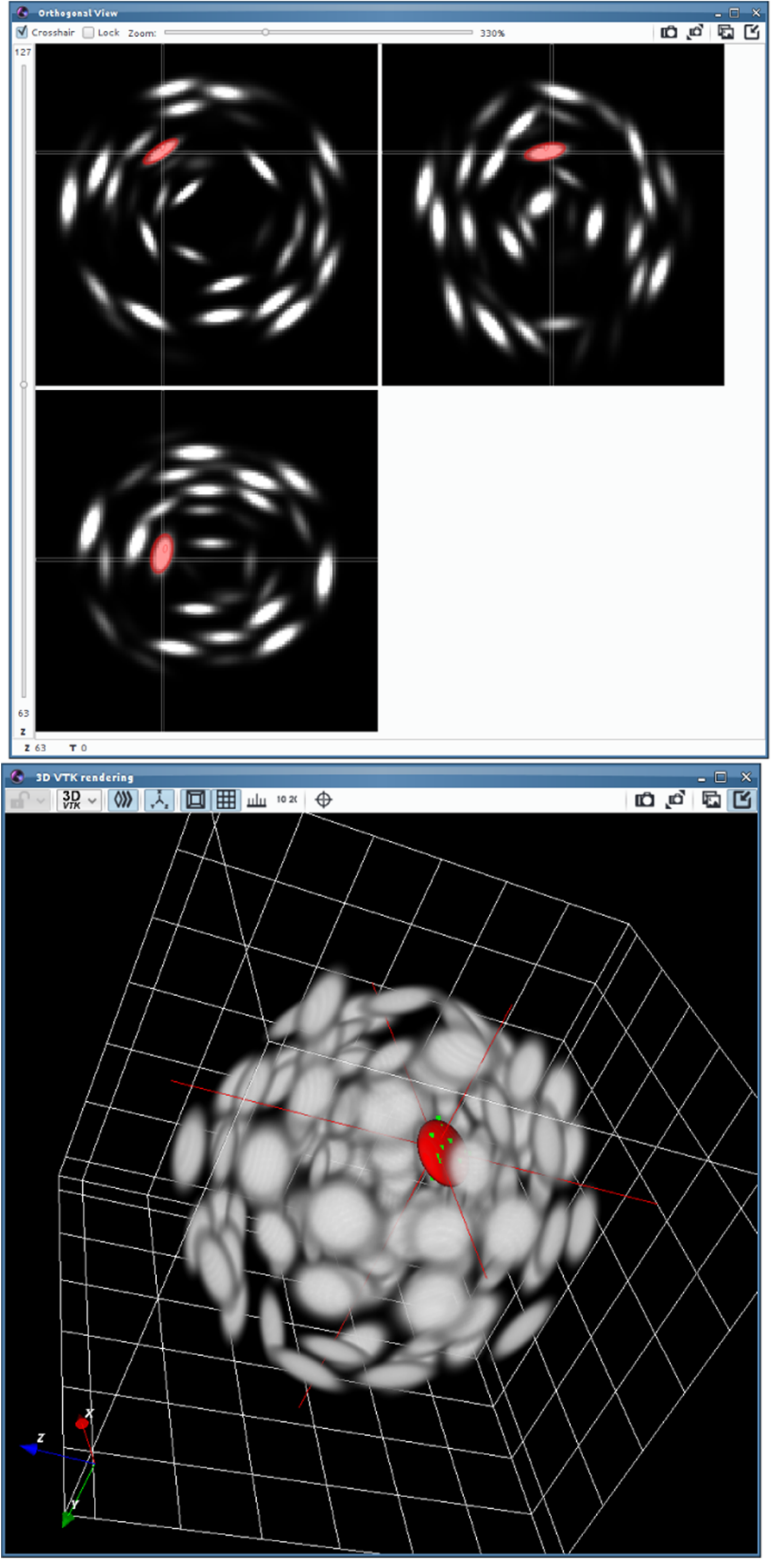
Fitting result on the synthetic 3D image of Fig. 1. Top: orthogonal views. Bottom: 3D rendering

Orthogonal views are probably the easiest way to interact with a 3D environment and their use is very common in biomedical imaging (see e.g. [29]). The user first selects a point in 3D space to define the 3 planes of interest and then locks the views to click on a few points on each plane. The operation can be repeated on multiple orthogonal views to sample the object surface more uniformly. When enough points have been selected, an algorithm described in the next section fits an ellipsoid to the point cloud. The operation can be repeated in the case when multiple ellipsoids have to be fitted. The result obtained by the point selection from Fig. 1 is displayed in Fig. 2.

Apart from the 3D visualization, the ellipsoids parameters (center, axes orientations and length of axes) are saved in a CSV file that can be read using standard spreadsheets or scientific computing softwares. In addition, it is possible to save a 3D binary image indicating the interior of each ellipsoid.

### Tutorial

A video tutorial is provided here http://youtu.be/MjotgTZi6RQ. It describes the main features of the plugin.

### Mathematical description

Given a set of *n* points *X*=(*x*_*i*_)_1≤*i*≤*n*_ in **R**^*d*^, where *d*=2 or 3, the objective of this section is to describe a fast and robust algorithm to fit an ellipsoid to those points. This is a longstanding problem studied in more than 40 journal papers. We refer to the book [25] for a more comprehensive overview. Two main approaches have been proposed to solve it.

**The geometric approach** This method was proposed in [13, 16, 22]. It consists in finding an ellipsoid *E* that minimizes the following least squares problem:

1 $$ F(E)=\sum\limits_{i=1}^{n} \text{dist}(x_{i},E)^{2},  $$

where dist(*x*_*i*_,*E*)= inf*x*∈*E*∥*x*−*x*_*i*_∥ is the Euclidean distance from the point *x*_*i*_ to the ellipsoid *E*. While this formulation has a clear geometrical meaning, it suffers from being highly nonconvex. Designing global minimization methods is therefore heavy from a computational point of view.

**The algebraic approach** This method is the one adopted in this paper. An ellipsoid *E* can be represented by a triplet (*A*,*b*,*c*) through an implicit equation of the form

2 $$ E = \left\{x \in \mathbf{R}^{d}, \langle x,Ax\rangle + \langle b,x\rangle +c=0 \right\},  $$

where *A*∈**R**^*d*×*d*^ is a symmetric positive definite matrix, *b*∈**R**^*d*^ is a vector and *c*∈**R** is a scalar.

The algebraic approach consists in minimizing the following residual

3 $$ G(X,A,b,c) = \sum\limits_{i=1}^{n} (\langle x_{i},{Ax}_{i}\rangle + \langle b,x_{i}\rangle +c)^{2},  $$

over a set $\mathcal {M}$ of admissible triplets (*A*,*b*,*c*). The sole positive definiteness condition *A*≻0 is not sufficient since the infimum of *G* over the set of positive semi-definite matrices is (*A*,*b*,*c*)=(0,0,0). It is necessary to add a normalization condition to avoid the trivial solution. Various possibilities have been considered in the literature. We follow the approach proposed in [17] that consists in imposing Tr(*A*)=1. This choice has the advantage of leading to a convex constraint, allowing to design efficient numerical algorithms. Overall, the optimization problem considered here reads

4 $$ \min_{(A,b,c) \in \mathcal{M}} G(X,A,b,c),  $$

where $\mathcal {M}=\left \{(A,b,c)\in \mathbf {R}^{d\times d}\times \mathbf {R}^{d} \times \mathbf {R}, A\succeq 0, \text {Tr}(A)=1\right \}$.

**Fig. 3:**
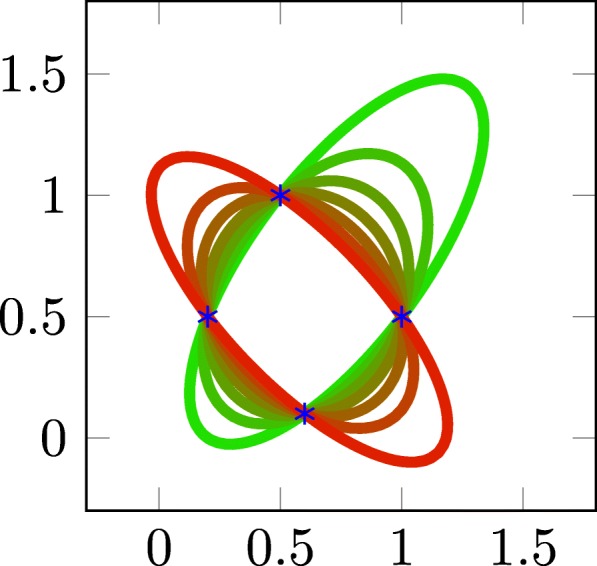
A family of ellipses passing through 4 points. In dimension *d*=2 the minimum number of points necessary to ensure uniqueness is *n*=5

The interests of this specific formulation are the following:

∙ There exists at least one minimizer. Moreover if the number of points *n* satisfies *n*≥*d*(*d*+1)/2+*d* and the points are in generic position, then the minimizer is unique, see Fig. 3 in 2D for an illustration and the Additional file 1 for a proof.

∙ The minimizer is covariant to translation and rotation of the input point locations *X*. More precisely, let $\hat E$ denote the ellipsoid solution of (4) and $\hat E'$ denote the ellipsoid obtained by solving (4) with input coordinates *X*^′^=(*xi*′)_1≤*i*≤*n*_, where *xi*′=*Rx*_*i*_+*t*, *R*∈**R**^*d*×*d*^ is a rotation matrix and *t*∈**R**^*d*^ is a translation vector. Then $\hat E'=R\hat E + t$. The proof of this property is detailed in the Additional file 1.

### A numerical algorithm

In [17], Calafiore suggested reformulating (4) as a semi-definite program and using interior point type methods to solve it. This type of algorithm is known to be robust and reliable but is rather hard to implement. Moreover, common primal-dual interior point methods [23] have a complexity that does not scale well with the number of input data points *n*. Based on this observation, Lin and Huang [26] designed a method based on the alternating direction method of multipliers (ADMM) to solve problem (4). While the per-iteration complexity of this approach is lower than that of interior point methods, the number of iterations is hard to control from a theoretical point of view, and we will show through numerical experiments that it can be very large to yield satisfactory solutions. We propose a more robust approach in what follows.

In 2D, the fact that the point *x*_*i*_ belongs to an ellipse represented by (*A*,*b*,*c*) can be rewritten in the compact form (see e.g. [13]):

$$\langle d_{i},q \rangle=0,$$

where

$$\begin{array}{*{20}l} d_{i}&=\left(x_{i}[1]^{2},x_{i}[2]^{2},\sqrt{2}x_{i}[1]x_{i}[2],x_{i}[1],x_{i}[2],1\right)^{T}, \\ q&=\left(a_{1,1},a_{2,2},\sqrt{2}a_{1,2},b_{1},b_{2},c\right)^{T}, \end{array} $$

and we denote *x*_*i*_[*j*] the *j*-th coordinate of the point *x*_*i*_. Now, letting *D*=[*d*_1_,…,*d*_*n*_], the objective function *G* can be rewritten as

5 $$ G(X,q) = \|D^{T} q \|^{2}.  $$

In 3D, a similar decomposition can be performed, see details in the Additional file 1.

Let *m*=*d*(*d*+1)/2+*d*+1 denote the number of parameters in *q*. The set of admissible vectors $\mathcal {Q}$ is defined as

6 $$ \mathcal{Q}=\left\{q\in \mathbf{R}^{m}, \text{Tr}(\mathcal{A}(q))=1, \mathcal{A}(q) \succeq 0\right\},  $$

where $\mathcal {A}:\mathbf {R}^{m} \to \mathbf {R}^{d\times d}$ is the linear mapping that associates matrix *A* to vector *q*. With the proposed notation, problem (4) simplifies to the following convex problem:

7 $$ \min_{q\in \mathcal{Q}} \|D^{T} q \|^{2}.  $$

We solve (7) using the Douglas-Rachford algorithm, which was first proposed by Lions and Mercier [30]. The details are presented in the Additional file 1.

### Invariance to affine transformations

#### Non invariance of the Algorithm

As discussed above, the minimizers of (7) are covariant to isometries. However the algorithm is not, this is illustrated in the Additional file 1. Moreover, the solutions of (4) are not invariant to affine transforms, which would be a desirable property. We propose to address both issues below. Similar ideas were proposed in [15] for the specific case of spheres.

#### Ensuring invariance using the SVD

In order to ensure invariance of the algorithm we change the coordinate system and work with a point cloud that is centered with covariance matrix equal to the identity. We obtain an ellipsoid in the modified system and finally map it back to the original one. This can be achieved using a singular value decomposition, as explained in the Additional file 1.

## Results

### Performance of the optimization algorithm

We report in the Additional file 1 experiments and comparisons on 2D data, as well as a robustness to noise study in 3D. We show that our numerical approach never requires more than 200 cheap iterations to reach machine precision, while the unnormalized method can require arbitrarily large computing times depending on the points set location. In addition, we provide comparisons with the simpler LLS algorithm [14] and show an improved robustness to noise.

### Segmentation experiments on synthetic data

**Fig. 4:**
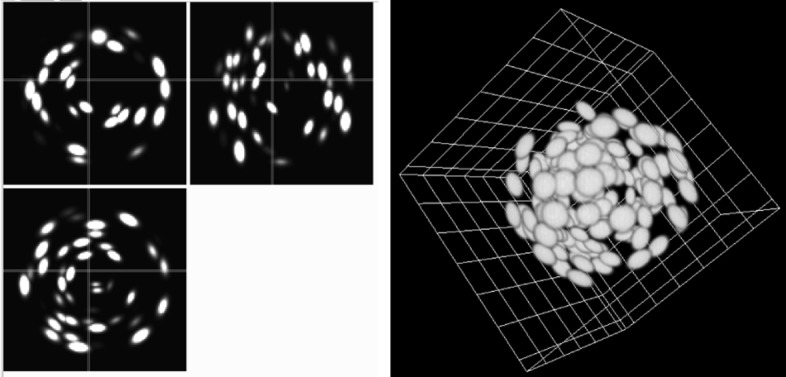
Synthetic spheroid used to assess the plugin’s accuracy. Left: the 3 orthogonal views, Right: 3D rendering

In order to assess the plugin’s efficiency in terms of: accuracy, reproducibility and time of user’s interaction, we designed a synthetic 3D image composed of 145 oblate^1^ ellipsoids mimicking a tumor spheroid, see Fig. 4. This image can be reproduced using the codes available on GitHub. The image was blurred with a Gaussian kernel of standard deviation equal to 1.5 pixel, to mimick what happens on a real microscope. Three users were asked to segment all the ellipsoids, and to time their task. The segmentation results were then compared with the ground truth. The results are displayed in Table 1. The column labelled ’time’ displays the average time spent by the user (in seconds) to segment one ellipsoid, the column ’center’ provides the average error (in pixel) between the true location of the center and the estimated one, the column ’angle’ corresponds to the average error (in degrees) of the orientation of the minor axis, and the column ’length’ corresponds to the average error (in pixels) on the 3 axes lengths.

**Table 1:** Segmentation time and accuracy for 3 different users

	Time (s)	Center (px)	Angle (deg)	Length (px)
User 1	25.7 ± 3.1	1.08 ± 0.32	4.6 ± 1.5	1.07 ± 0.41
User 2	26.1 ± 5.0	0.90 ± 0.35	5.9 ± 2.5	0.54 ± 0.35
User 3	20.9 ± 3.3	1.01 ± 0.36	4.9 ± 1.7	0.68 ± 0.36

The number after the sign ± represents the standard deviation

Notice that the accuracy on the center location and on the axis length is below the resolution of 1.5 pixel. It is then possible to claim that our plugin allows to obtain subresolution results for perfectly ellipsoidal objects in a few seconds. Note that this time is the time required for the user to select the points, the computation time is in fractions of a second. In addition, the angular accuracy is also satisfactory, suggesting that the plugin can be used to analyze the geometry of large collections of objects.

### Segmentation experiments on real 3D tumor spheroids

**Fig. 5:**
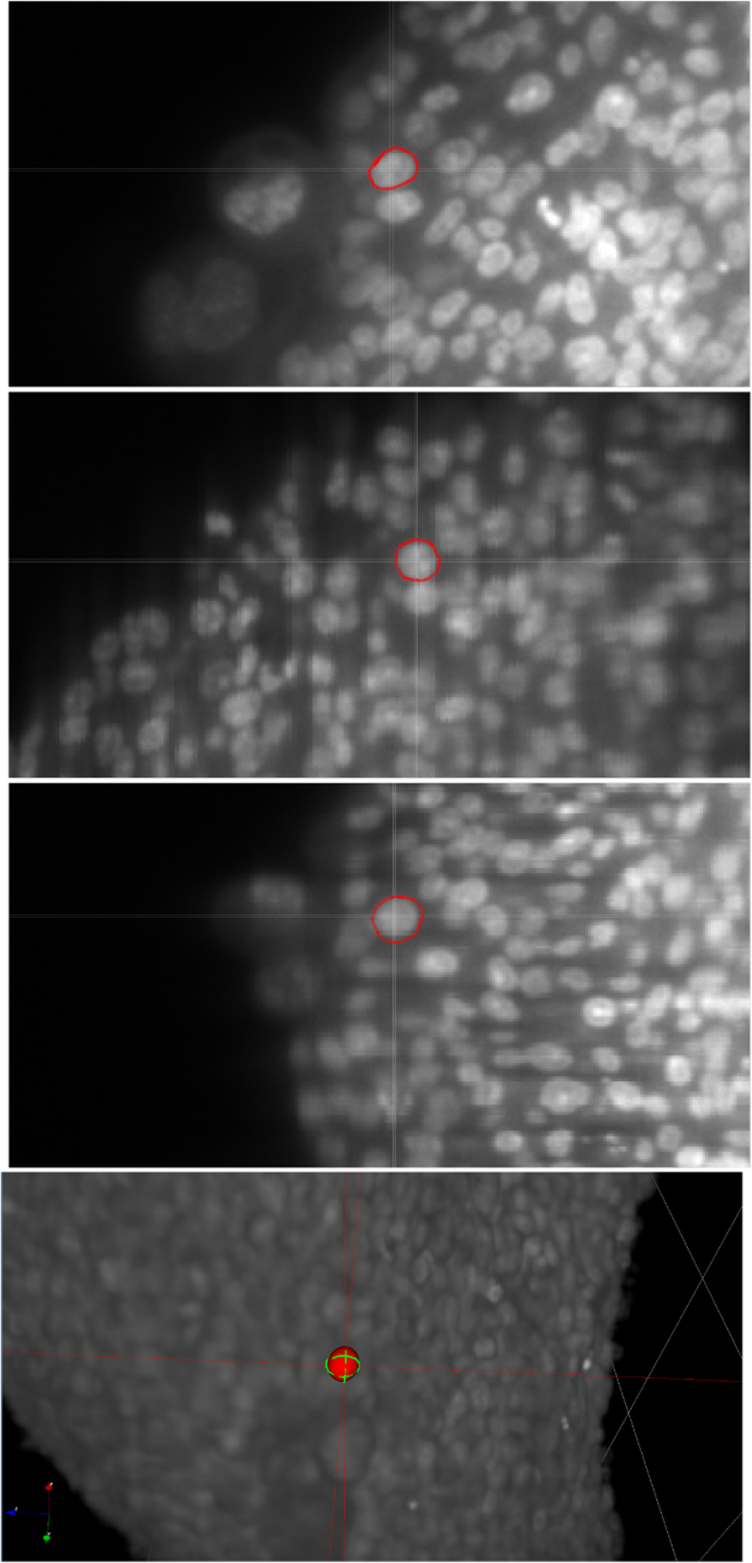
Segmentation of a cell nucleus on a real 3D image. From top to bottom: the points selected on the 3 orthogonal views, 3D rendering of the result of the segmentation

**Fig. 6:**
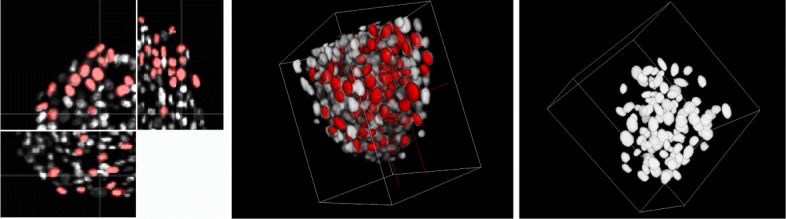
Segmentation of cell nuclei on a real 3D image. From left to right: the segmented ellipses on the 3 orthogonal views, 3D rendering of the result of the segmentation, 3D rendering of the binary image provided by the plugin

The plugin FitEllipsoid was used to segment cell nuclei in spheroids. We show in Figs. 5 and 6 two examples of 3D tumor spheroids. The one in Fig. 5 is a spheroid with a large diameter of 500 microns leading to a poor image quality due to light scattering and absorption. Figure 6 presents a smaller spheroid with a diameter of 150 microns.

The biological question we addressed was to estimate the distribution of shapes of nuclei, through the estimation of their semi-axes lengths *ℓ*_1_≥*ℓ*_2_≥*ℓ*_3_. Two different experimental conditions have been explored: untreated freely-grown spheroids and spheroids treated for 8h with Latrunculin, a drug targetting actin cytoskeleton.

**Fig. 7:**
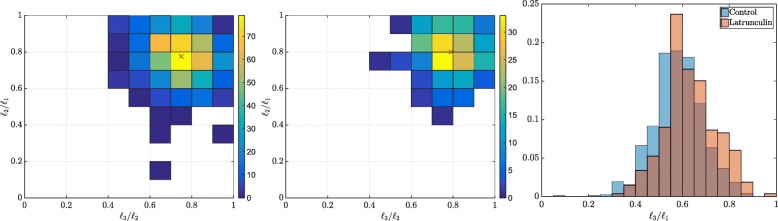
Analysis of the nuclei geometry for control and Latrunculin treated spheroids. Left and center: 2D histograms representing the ratio *ℓ*_2_/*ℓ*_1_ vs *ℓ*_3_/*ℓ*_2_ for the control (left) and the treated spheroids (center). The red cross indicates the mean of the distribution. Right: 1D histograms of the aspect ratio *ℓ*_3_/*ℓ*_1_ for the control and treated spheroids

We used FitEllipsoid to segment *n*=708 nuclei from *x*=19 control spheroids and *n*=266 nuclei from *x*=7 spheroids treated with Latrunculin. We display a 2D-histogram of the joint distribution *ℓ*_2_/*ℓ*_1_ vs *ℓ*_3_/*ℓ*_2_ for each condition in Fig. 7. A prolate spheroid (a rugby ball) satisfies *ℓ*_3_/*ℓ*_2_≃1 and *ℓ*_2_/*ℓ*_1_<1 and on this graph, it corresponds to a point on the right boundary of the unit square. An oblate spheroid on its side, satisfies *ℓ*_3_/*ℓ*_2_<1 and *ℓ*_2_/*ℓ*_1_≃1. It corresponds to a point on the top boundary of the square. The sphere coincides with *ℓ*_3_/*ℓ*_2_=*ℓ*_2_/*ℓ*_1_=1, which is the top-right corner.

On the histograms, we can observe that the distribution is denser along the diagonal, and that there is no clear trend towards a prolate or oblate shape. It is however clear that the nuclei are not spherical. The 1D histograms on the right of Fig. 7 shows that the aspect ratio (defined as *ℓ*_3_/*ℓ*_1_) of a nuclei is shifted towards 1 when going from the treated to the control spheroids. The average aspect ratio is 0.58 for the control spheroid and 0.63 for the Latrunculin treated spheroid.

Overall we see that the plugin allows to distinguish subtle but statistically significant changes of shapes.

## Conclusions

FitEllipsoid is a powerful tool for supervised ellipsoids segmentation, with a user-friendly interface. The computational part of the software is based on a novel algorithm that is invariant under affine transforms. It allowed to segment hundreds of cell nuclei in order to analyze statistically their shape.

## Additional file

Additional file 1 This file contains mathematical facts and proofs regarding our approach, the detailed algorithms, and quantitative 2D and 3D comparisons with other approaches. (PDF 496 kb)

## Abbreviations

ADMM: Alternating direction method of multipliers
CSV: Coma separated values
LLS: Linear least squares
SPIM: Single plane imaging microscope
VTK: Visualization ToolKit

## Footnotes

1 We used this geometry since we thought that it would correspond to what is observed in real tumor spheroids. We will see later that this is actually not the case.

## References

[1] Lockett SJ, Sudar D, Thompson CT, Pinkel D, Gray JW (1998). Efficient, interactive, and three-dimensional segmentation of cell nuclei in thick tissue sections. Cytometry.

[2] Okada K, Comaniciu D, Krishnan A (2005). Robust anisotropic gaussian fitting for volumetric characterization of pulmonary nodules in multislice ct. IEEE Trans Med Imaging.

[3] Mahdavi S, Salcudean SE (2008). 3d prostate segmentation based on ellipsoid fitting, image tapering and warping. Engineering in Medicine and Biology Society, 2008. EMBS 2008. 30th Annual International Conference of the IEEE.

[4] Dufour A, Shinin V, Tajbakhsh S, Guillén-Aghion N, Olivo-Marin J-C, Zimmer C (2005). Segmenting and tracking fluorescent cells in dynamic 3-d microscopy with coupled active surfaces. IEEE Trans Image Process.

[5] Thevenaz P, Delgado-Gonzalo R, Unser M (2011). The ovuscule. IEEE Trans Pattern Anal Mach Intell.

[6] Cuingnet R, Prevost R, Lesage D, Cohen LD, Mory B, Ardon R (2012). Automatic detection and segmentation of kidneys in 3d ct images using random forests. International Conference on Medical Image Computing and Computer-Assisted Intervention.

[7] Delgado-Gonzalo R, Chenouard N, Unser M (2013). Spline-based deforming ellipsoids for interactive 3d bioimage segmentation. IEEE Trans Image Process.

[8] Olivo-Marin J-C (2002). Extraction of spots in biological images using multiscale products. Pattern Recog.

[9] Jaqaman K, Loerke D, Mettlen M, Kuwata H, Grinstein S, Schmid SL, Danuser G (2008). Robust single-particle tracking in live-cell time-lapse sequences. Nat Methods.

[10] Soubies E, Weiss P, Descombes X (2013). A 3d segmentation algorithm for ellipsoidal shapes. application to nuclei extraction. ICPRAM-International Conference on Pattern Recognition Applications and Methods.

[11] Zhang W, Fehrenbach J, Desmaison A, Lobjois V, Ducommun B, Weiss P (2016). Structure tensor based analysis of cells and nuclei organization in tissues. IEEE Trans Med Imaging.

[12] De Chaumont F, Dallongeville S, Chenouard N, Hervé N, Pop S, Provoost T, Meas-Yedid V, Pankajakshan P, Lecomte T, Le Montagner Y (2012). Icy: an open bioimage informatics platform for extended reproducible research. Nat Methods.

[13] Gander W, Golub GH, Strebel R (1994). Least-squares fitting of circles and ellipses. BIT Numer Math.

[14] Fitzgibbon A, Pilu M, Fisher RB (1999). Direct least square fitting of ellipses. IEEE Trans Pattern Anal Mach Intell.

[15] Nievergelt Y (2001). Hyperspheres and hyperplanes fitted seamlessly by algebraic constrained total least-squares. Linear Algebra Appl.

[16] Ahn SJ, Rauh W, Warnecke H-J (2001). Least-squares orthogonal distances fitting of circle, sphere, ellipse, hyperbola, and parabola. Pattern Recogn.

[17] Calafiore G (2002). Approximation of n-dimensional data using spherical and ellipsoidal primitives. IEEE Trans Syst Man Cybern Syst Hum.

[18] Ahn SJ, Rauh W, Cho HS, Warnecke H-J (2002). Orthogonal distance fitting of implicit curves and surfaces. IEEE Trans Pattern Anal Mach Intell.

[19] Markovsky I, Kukush A, Van Huffel S (2004). Consistent least squares fitting of ellipsoids. Numer Math.

[20] Li Q, Griffiths JG (2004). Least squares ellipsoid specific fitting. Geometric Modeling and Processing, 2004. Proceedings.

[21] Chernov N, Lesort C (2005). Least squares fitting of circles. J Math Imaging Vis.

[22] Kleinsteuber M, Hüper K (2010). Approximate geometric ellipsoid fitting: A cg-approach. Recent Advances in Optimization and Its Applications in Engineering.

[23] Ying X, Yang L, Zha H (2012). A fast algorithm for multidimensional ellipsoid-specific fitting by minimizing a new defined vector norm of residuals using semidefinite programming. IEEE Trans Pattern Anal Mach Intell.

[24] Saunderson J, Chandrasekaran V, Parrilo PA, Willsky AS (2012). Diagonal and low-rank matrix decompositions, correlation matrices, and ellipsoid fitting. SIAM J Matrix Anal Appl.

[25] Kanatani K, Sugaya Y, Kanazawa Y (2016). Ellipse fitting for computer vision: implementation and applications. Synth Lect Comput Vis.

[26] Lin Z, Huang Y (2016). Fast multidimensional ellipsoid-specific fitting by alternating direction method of multipliers. IEEE Trans Pattern Anal Mach Intell.

[27] Matlab code repository. https://github.com/pierre-weiss/FitEllipsoid. Accessed 20 Feb 2019.

[28] Schroeder WJ, Lorensen B, Martin KM. The visualization toolkit: an object-oriented approach to 3D graphics. Kitware publisher. 2004. http://cds.cern.ch/record/798217/files/1930934122_TOC.pdf.

[29] Heller GV, Cerqueira MD, Weissman NJ, Dilsizian V, Jacobs AK, Kaul S, Laskey WK, Pennell DJ, Rumberger JA, Ryan T (2002). Standardized myocardial segmentation and nomenclature for tomographic imaging of the heart: a statement for healthcare professionals from the cardiac imaging committee of the council on clinical cardiology of the american heart association. J Nucl Cardiol.

[30] Lions P-L, Mercier B (1979). Splitting algorithms for the sum of two nonlinear operators. SIAM J Numer Anal.

