# Supplementary material for: FitEllipsoid: a fast supervised ellipsoid segmentation plugin
Source: BMC Bioinformatics. 2019 Mar 15;20:142. doi: 10.1186/s12859-019-2673-0 (PMC6419800; doi:10.1186/s12859-019-2673-0)
Supplement: Supplementary file 1 — This file contains mathematical facts and proofs regarding our approach, the detailed algorithms, and quantitative 2D and 3D comparisons with other approaches. (PDF 496 kb) [file 12859_2019_2673_MOESM1_ESM.pdf]

SUPPLEMENTARY MATERIAL

FitEllipsoid: a fast supervised ellipsoid segmentation plugin

Bastien Kovac[1], Jérôme Fehrenbach[1,2*], Ludivine Guillaume[1] and Pierre Weiss[1,2]

In this supplementary file we provide details regarding the proposed algorithm. We derive a few theoretical results, we present comparisons with other algorithms in 2D and illustrate the robustness to noise in 3D.

Mathematical approach

Reformulation of (4) into (7)

In 2D, the fact that point x_i belongs to an ellipse represented by (A, b, c) reads:

$$a_{1,1}x_i[1]^2 + a_{2,2}x_i[2]^2 + 2a_{1,2}x_i[1]x_i[2] + b_1x_i[1] + b_2x_i[2] + c = 0. \quad (8)$$

By stacking the coefficients in a vector

$$q = (a_{1,1}, a_{2,2}, \sqrt{2}a_{1,2}, b_1, b_2, c)^T,$$

equation (8) can be rewritten in the compact form (see e.g. [1])

$$\langle d_i, q\rangle = 0,$$

where

$$d_i = (x_i[1]^2, x_i[2]^2, \sqrt{2}x_i[1]x_i[2], x_i[1], x_i[2], 1)^T. \quad (9)$$

Now, letting $D = [d_1, \ldots, d_n]$, function G can be rewritten as

$$G(X, q) = \|D^T q\|^2. \quad (10)$$

In 3D, a similar decomposition can be performed. We used the following conventions in our codes

$$q = (a_{1,1}, a_{2,2}, a_{3,3}, \sqrt{2}a_{1,2}, \sqrt{2}a_{1,3}, \sqrt{2}a_{2,3}, b_1, b_2, b_3, c)^T,$$

$$d_i = (x_i[1]^2, x_i[2]^2, x_i[3]^2, \sqrt{2}x_i[1]x_i[2], \sqrt{2}x_i[1]x_i[3], \sqrt{2}x_i[2]x_i[3], x_i[1], x_i[2], x_i[3], 1)^T. \quad (11)$$

*Correspondence: jerome.fehrenbach@math.univ-toulouse.fr
[1]ITAV, CNRS, Université de Toulouse, 1 Pl. Pierre Potier, 31106 Toulouse, France
Full list of author information is available at the end of the article

Let $m = d(d+1)/2 + d + 1$ denote the number of parameters in q. The set of admissible vectors $\mathcal{Q}$ is then defined as:

$$\mathcal{Q} = \{q \in \mathbf{R}^m, \mathrm{Tr}(\mathcal{A}(q)) = 1, \mathcal{A}(q) \succeq 0\}, \quad (12)$$

where $\mathcal{A} : \mathbf{R}^m \to \mathbf{R}^{d\times d}$ is the linear mapping that associates matrix A to vector q. With the proposed notation, problem (4) simplifies to the following convex problem:

$$\min_{q\in\mathcal{Q}} \|D^T q\|^2. \quad (13)$$

Minimizers and invariance

Proposition 1 *Problem (4) is convex. It admits at least one minimizer. Set*

$$m = d(d+1)/2 + d + 1. \quad (14)$$

If $n \le m-2$, the minimizer is non unique. If the points are in "generic" position and the number $n \ge m-1$, then the minimizer is unique.

Proof We consider the equivalent formulation (7) instead of (4).

Problem (7) is convex since $\mathcal{Q}$ and $q \mapsto \|D^T q\|^2$ are both convex. It consists of a projection problem on $\mathcal{Q}$ with the possibly degenerate metric $q \mapsto \|D^T q\|^2$. Standard convex analysis results [2, Prop. 2.3.4] state that the solution exists and it is unique when the metric is non degenerate.

If $n \le m-2$, notice that $\dim(\ker(D^T)) \ge 2$ and $\dim(\{q \in \mathbf{R}^m, \mathrm{Tr}(\mathcal{A}(q)) = 1\}) = m-1$. Hence, there is a subspace V of dimension at least 1 such that $\forall q \in V$, $D^T q = 0$ and $\mathrm{Tr}(\mathcal{A}(q)) = 1$. All vectors q in this subspace satisfy $\|D^T q\|_2^2 = 0$, hence they are solution of (7). They all describe ellipsoids passing perfectly through the set of points.

If $n \ge m-1$, the genericity hypothesis amounts to assume that $\ker(D^T))$ is of dimension less or equal than 1 and is transverse to $\{q \in \mathbf{R}^m, \mathrm{Tr}(\mathcal{A}(q)) = 1\}$. Hence, function $q \mapsto \frac{1}{2}\|D^T q\|_2^2$ is strictly convex over $\{q \in \mathbf{R}^m, \mathrm{Tr}(\mathcal{A}(q)) = 1\}$ ensuring uniqueness of the minimizer. □

Remark 1 *An ellipsoid should satisfy $A \succ 0$ and not $A \succeq 0$. However, it is important to work over closed sets to ensure existence of a minimizer. The minimizer of (4) can therefore represent a degenerate ellipsoid such as a line in 2D or a plane in 3D. This situation never happened in our numerical experiments.*

Proposition 2 *The minimizer $(\hat{A}, \hat{b}, \hat{c})$ is covariant to translation and rotation of the input point locations X. More precisely, let $\hat{E}$ denote the ellipsoid solution of (4) and $\hat{E}'$ denote the ellipsoid obtained by solving (4) with input coordinates $X' = (x'_i)_{1\leq i\leq n}$, where $x'_i = Rx_i + t$, $R \in \mathbf{R}^{d\times d}$ is an orthogonal matrix and $t \in \mathbf{R}^d$ is a translation vector. Then $\hat{E}' = R\hat{E} + t$.*

Proof Let E denote an ellipsoid defined through the triplet $(A, b, c) \in \mathcal{M}$. Now, let $E' = RE + t$. A change of variable shows that E' is defined through (A', b', c') with

$$A' = RAR^T \ ,\ b' = Rb - 2A't \text{ and } c' = c - \langle t, A't + b'\rangle. \quad (15)$$

In addition, it is clear that $(A', b', c') \in \mathcal{M}$ since the trace and eigenvalues are invariant to isometries.

Straightforward calculus (using the relationship $R^T R = RR^T = I_d$) shows that

$$G(X, A, b, c) = G(X', A', b', c'). \quad (16)$$

Hence the minimizers $(\hat{A}, \hat{b}, \hat{c})$ of $G(X, A, b, c)$ over $\mathcal{M}$ and the minimizer $(\hat{A}', \hat{b}', \hat{c}')$ of $G(X', A', b', c')$ are related through (15). □

Remark 2 *The solution of (4) is however not covariant to affine changes. In the case of an affine change of coordinate, the orthogonality identity $R^T R = I_d$ used in the proof is not satisfied anymore.*

Algorithmic approach

We propose solving (7) using Douglas-Rachford algorithm which was first proposed by Lions and Mercier [3]. It is a simple algorithm to solve problems of the following type:

$$\min_{q\in\mathbf{R}^m} f_1(q) + f_2(q), \quad (17)$$

where $f_1 : \mathbf{R}^m \to \mathbf{R}\cup\{+\infty\}$ and $f_2 : \mathbf{R}^m \to \mathbf{R}\cup\{+\infty\}$ are extended real-valued convex closed functions such that $f_1(x) + f_2(x) \to +\infty$ as $\|x\| \to +\infty$. It is described in Algorithm 1, while Proposition 3 states its convergence properties. We recall that the proximal operator of a function f is defined by:

$$\mathrm{Prox}_{\gamma f}(z) = \operatorname*{argmin}_{x\in\mathbf{R}^n} \gamma f(x) + \frac{1}{2}\|x - z\|^2.$$

Algorithm 1 Douglas-Rachford algorithm to minimize (17)

Input: Initial guess $q^{(1/2)} \in \mathbf{R}^m$, number of iterations Nit, parameter $\gamma > 0$.
Output: $q^{(Nit)}$ an approximate solution of (17).
for $k = 1$ to Nit **do**

$$q^{(k)} := \mathrm{Prox}_{\gamma f_2}(q^{(k-1/2)})$$
$$q^{(k+1/2)} := q^{(k-1/2)} - q^{(k)} + \mathrm{Prox}_{\gamma f_1}(2q^{(k)} - q^{(k-1/2)}).$$

end for

Proposition 3 (Convergence [3, 4]) *The sequence $(q^{(k)})_{k\in\mathbf{N}}$ converges to a solution of (17).*

To apply this algorithm to (7), we set

$$f_1(q) = \|D^T q\|^2 \quad (18)$$

and

$$f_2(q) = \begin{cases} 0 & \text{if } q \in \mathcal{Q}, \\ +\infty & \text{otherwise.} \end{cases}$$

It now suffices to evaluate the proximal operators of f_1 and f_2. They are given in proposition 4 and 5 below.

Proposition 4 *The proximal operator of f_1 is given by*

$$\mathrm{Prox}_{\gamma f_1}(z) = (\gamma DD^T + \mathrm{Id})^{-1}(z). \quad (19)$$

Proposition 5 *Let $z \in \mathbf{R}^m$. Assume that $\mathcal{A}(z)$ can be diagonalized as $\mathcal{A}(z) = U\Sigma U^T$, where $\Sigma = \mathrm{diag}(\sigma)$. Let σ_+ denote the projection of σ on the unit simplex and define $A_+ = U\mathrm{diag}(\sigma_+)U^T$. Then $\mathrm{Prox}_{\gamma f_2}(z)$ is obtained by changing the first $d(d+1)/2$ components of z by $\mathcal{A}^{-1}(A_+)$ and leaving the others unchanged.*

Proof It suffices to note that:

$$\begin{aligned} &\min_{\mathcal{A}(q)\succeq 0, \mathrm{Tr}(\mathcal{A}(q))=1} \frac{1}{2}\|q - z\|^2 \\ &= \min_{\mathcal{A}(q)\succeq 0, \mathrm{Tr}(\mathcal{A}(q))=1} \frac{1}{2}\|\mathcal{A}(q) - \mathcal{A}(z)\|_F^2 \\ &= \min_{S\succeq 0, \mathrm{Tr}(S)=1} \frac{1}{2}\|S - \Sigma\|_F^2. \end{aligned}$$

where $\|\cdot\|_F$ denotes the Frobenius norm, which is invariant by unitary transforms. Projecting a vector on the unit simplex is a standard issue, see e.g. [5]. It can be solved exactly in $O(d)$ operations. □

Remark 3 *In [6], the authors proposed using the ADMM algorithm [7], which can be seen as Douglas-Rachford algorithm applied to the dual of* (17). *Their implementation relies on the fact that* $\mathcal{Q} = \mathcal{Q}_1 \cap \mathcal{Q}_2$, *where* $\mathcal{Q}_1$ *describes the set of symmetric positive semi-definite matrices and* $\mathcal{Q}_2$ *describes the set of matrices with trace equal to* 1. *They then propose to split the problem in three terms (one for* $\mathcal{Q}_1$, *one for* $\mathcal{Q}_2$ *and one for* f_1*) while our decomposition uses only two terms. This simplifies the algorithm by reducing the number of parameters to tune to* 1*: the value of* γ.

An effective algorithm

The algorithm to solve the ellipsoid fitting problem (4) is given in Algorithm 2. It depends on two extra parameters: the number of iterations Nit and a value $\gamma > 0$. The number of iterations to achieve a reasonable result strongly depends on normalizing conditions described in the next section. The convergence is also sensitive to the value of γ. However, with the normalization proposed in the next section, it can be tuned once for all. In all our numerical experiments, we simply set $\gamma = 10$.

Algorithm 2 An algorithm to solve (4)

Input: Data points $X = [x_1, \ldots, x_n]$, Number of iterations Nit, Parameter $\gamma > 0$.
Output: The parameters q of an ellipsoid.
Compute matrix D using equation (9) in 2D or (11) in 3D.
Set $c_i = \mathrm{mean}(X(:,i))$.
Set $r^2 = \mathrm{mean}(\sum_{i=1}^d (X(:,i) - c_i)^2)$.
if d=2 **then**
  Set $q^{(1/2)} = [0.5, 0.5, 0, -c_1, -c_2, (c_1^2 + c_2^2 - r^2)/2]$.
else if d=3 **then**
  Set $q^{(1/2)} = [0.5, 0.5, 0.5, 0, 0, 0, -c_1, -c_2, -c_3, (c_1^2 + c_2^2 + c_3^2 - r^2)/2]$.
end if
Call Algorithm 1, with f_1 defined in (18).

We observed that the algorithm performance strongly depends on the points locations. This is illustrated in Fig. 1 for coordinate shifts and in Fig. 2 for the dilation of one axis. In addition, the solutions of (4) are not invariant to affine transforms, which is a desirable property. We propose to address both issues below. Similar ideas were proposed in [8] for the specific case of spheres.

Let $X = [x_1, \ldots, x_n] \in \mathbf{R}^{d\times n}$. We are looking for a linear transform $P \in \mathbf{R}^{d\times d}$ and a translation vector $t \in \mathbf{R}^d$, such that the vectors

$$y_i = P(x_i - t) \tag{20}$$

have mean 0 and covariance matrix Id. Letting $Y = [y_1, \ldots, y_n]$, this means that $YY^T = \mathrm{Id}$.

For centering X, we clearly need to take

$$t = \frac{1}{n}\sum_{i=1}^n x_i. \tag{21}$$

Now, let $\bar{X} = [x_1 - t, \ldots, x_n - t]$ denote the set of centered vectors. The eigenvalue decomposition of $\bar{X}\bar{X}^T$ reads

$$\bar{X}\bar{X}^T = \bar{U}\bar{\Sigma}\bar{U}^T. \tag{22}$$

By letting

$$P = \Sigma^{-1/2}U^T, \tag{23}$$

we obtain the desired result.

Algorithm 3 summarizes the proposed idea. It strongly improves the algorithm's convergence. In practice, this approach never required more than 200 iterations to reach machine precision, while the unnormalized method can require arbitrarily large computing times depending on the points set location.

Algorithm 3 Ellipsoid fitting using SVD normalization

Input: Data points X.
Output: An ellipsoid E.
Evaluate t and P using (21), (22) and (23).
Construct $y_i = P(x_i - t)$.
Apply Algorithm 2 with input $Y = [y_1, \ldots, y_n]$, $Nit = 100$ and $\gamma = 10$.
Set $E = P^{-1}E + t$.

Comparison in 2D

In this section we illustrate the behavior of different algorithms on a 2D example that is representative of numerous experiments. We compare four algorithms both in terms of computing times and robustness of the results with respect to i) noise and ii) non-uniform sampling. In all experiments, the same ellipse is used. Its center is set to $z = (4, 5)$ and the lengths of its axes are 4 and 1.

1. The first algorithm denoted DR is described in Algorithm 2.
2. The second algorithm denoted DR-SVD is Algorithm 3.
3. The third algorithm is the standard Linear Least Square approach [10] denoted LLS. It consists in choosing the normalization condition $\|q\| = 1$ in the minimization of G given by Equation (3The algebraic approachequation.0.3). When skipping the constraint $\mathcal{A}(q) \succeq 0$, the problem becomes

$$\min_{\|q\|=1} \|D^T q\|^2, \tag{24}$$

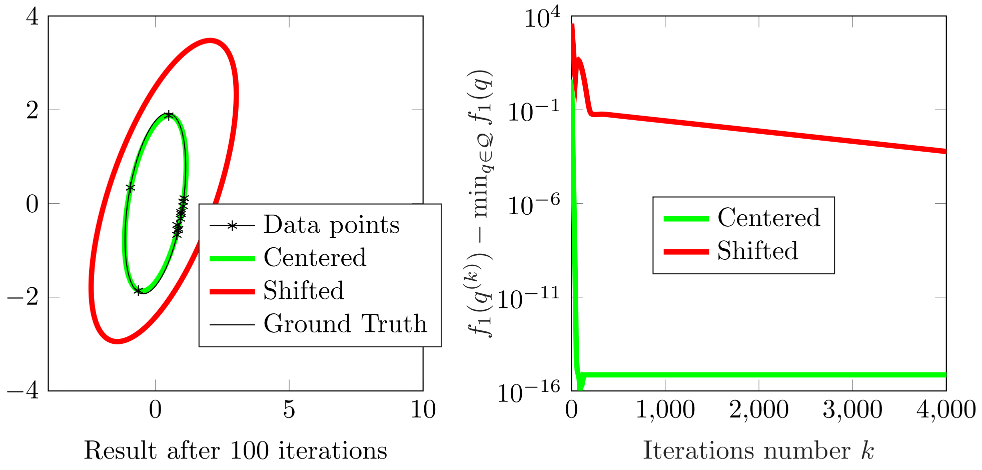

Figure 1 Shift experiment: in this experiment, we fit the set of points indicated with asterisks using 100 iterations of Douglas-Rachford algorithm 1, or the same set shifted by 10 units horizontally. We then plot the result at the same origin. As can be seen, the green ellipsoid (non shifted points) is well retrieved, while the one associated to shifted points is unsatisfactory. The same behavior can be observed for the cost function. The convergence curve associated to the non shifted points shows that machine precision is reached after about 50 iterations. On its side, the convergence curve associated to the shifted points converges linearly with a very slow rate. It reaches the maximal reachable precision (10^{-10}) in about 20,000 iterations.

which amounts to finding the smallest eigenvector of $K = DD^T$. It yields a vector $\tilde{q}$ that describes a quadric, which is not necessarily an ellipsoid.

4 The fourth algorithm denoted LLS-SVD consists of applying LLS to the dataset after a change of coordinates, similarly to what is described in Algorithm 3. This algorithm is affine invariant.

In Figure 3 (left) one can observe that all algorithms provide satisfactory results, although DR-SVD is faster than DR. Note that LLS and LLS-DR are very rapid since they consist of finding the smallest eigenvector of a 6×6 matrix. In Figure 3 (right) the situation is different since the points are not sampled regularly along the ellipse. In this situation, the solutions provided by LLS and LLS-SVD are not ellipses, this comes from the fact that the constraint of positivity of the matrix $A(q)$ is not included in the LLS formulation of the problem.

3D experiments

In this section, we perform a few experiments to challenge the algorithm implemented in the Icy plugin. To do so, we draw points uniformly at random on the boundary of an ellipsoid on 3 orthogonal planes. We then add a random perturbation of normal distribution with variance σ^2 within each plane. This way, we simulate what a user does by clicking on points in 3 orthogonal views. Note that the orthogonal planes orientation do not necessarily coincide with the ellipsoid axes.

Minimal amount of points

The objective here is to illustrate the minimal number of points required and the stability to noise. As can be seen in this example, 20 points are enough to provide an accurate result despite a significant amount of noise.

Effect of the SVD on the precision

We saw in the previous paragraph that the SVD scaling allows to strongly improve the convergence speed of the iterative algorithm. In this section, we illustrate the effect of the SVD scaling on the precision of the estimate. We draw 100 ellipsoids with random orientations with an oblate shape ($l_1 = 1$, $l_2 = 0.5$, $l_3 = 0.5$) or a prolate shape ($l_1 = 1$, $l_2 = 1$, $l_3 = 0.5$). For each of these, we draw 8 points randomly on the boundary of the ellipsoid intersected with each plane XY, XZ and YZ. This yields 24 points in total. We then add random Gaussian noise with various standard deviations $\sigma \in \{0, 0.05, 0.1\}$. This is in the range of what an end-user could do. We estimate the ellipsoids with and without the SVD scaling and report the Haudorff distance between the true ellipsoid and the estimated ellipsoid in Table 1. As can be seen, the SVD scaling slightly deteriorates the estimation quality, but the results are overall on par.

Author details
[1]ITAV, CNRS, Université de Toulouse, 1 Pl. Pierre Potier, 31106 Toulouse, France. [2]IMT, CNRS, Université de Toulouse, 118, route de Narbonne, 31400 Toulouse, France.

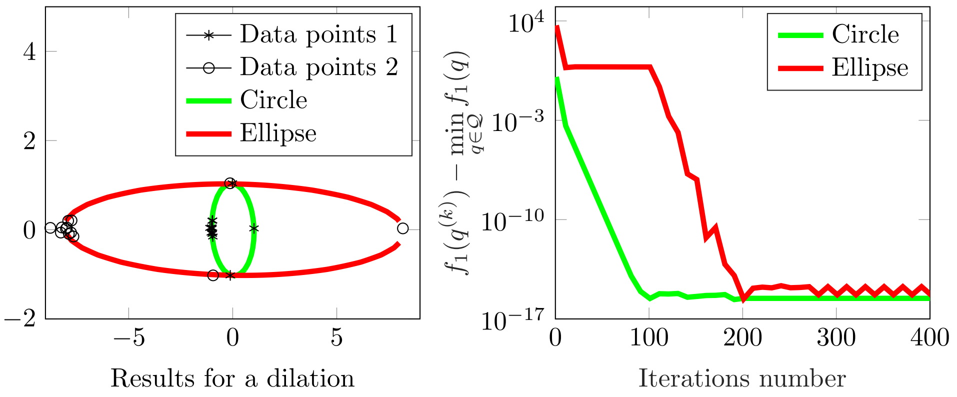

Figure 2 Linear transform experiment: in this experiment, we fit the set of points indicated with asterisks using 400 iterations of Algorithm 1, or the same set of points dilated by a factor 8 along the x-axis, see left Figure. The convergence curves show different behaviors: while the curve associated to the circle shows a favorable linear behavior right from the start, the curve associated to the ellipse has a plateau for the first 110 iterations and then then has the same favorable linear behavior. This phenomenon illustrates recent results about Douglas-Rachford algorithm dynamics [9]: the algorithm converges to a manifold in a finite number of iterations and then shows a linear convergence rate.

Table 1 Segmentation time and accuracy for 3 different users. The number after the sign $\pm$ represents the standard deviation.

	Prolate		Oblate	
	Without	With	Without	With
$\sigma = 0$	0	0	0	0
$\sigma = 0.05$	0.074 $\pm$ 0.037	0.098 $\pm$ 0.75	0.093 $\pm$ 0.051	0.096 $\pm$ 0.071
$\sigma = 0.1$	0.207 $\pm$ 0.153	0.217 $\pm$ 0.134	0.156 $\pm$ 0.069	0.169 $\pm$ 0.079

References

1. Gander, W., Golub, G.H., Strebel, R.: Least-squares fitting of circles and ellipses. BIT Numerical Mathematics **34**(4), 558–578 (1994)
2. Bertsekas, D.P., Nedi, A., Ozdaglar, A.E., et al.: Convex analysis and optimization (2003)
3. Lions, P.-L., Mercier, B.: Splitting algorithms for the sum of two nonlinear operators. SIAM Journal on Numerical Analysis **16**(6), 964–979 (1979)
4. Combettes, P.L., Pesquet, J.-C.: Proximal splitting methods in signal processing. In: Fixed-point Algorithms for Inverse Problems in Science and Engineering, pp. 185–212. Springer, ??? (2011)
5. Condat, L.: Fast projection onto the simplex and the\ pmb {l} _\ mathbf {1} ball. Mathematical Programming **158**(1-2), 575–585 (2016)
6. Lin, Z., Huang, Y.: Fast multidimensional ellipsoid-specific fitting by alternating direction method of multipliers. IEEE transactions on pattern analysis and machine intelligence **38**(5), 1021–1026 (2016)
7. Glowinski, R., Marroco, A.: Sur l'approximation, par éléments finis d'ordre un, et la résolution, par pénalisation-dualité d'une classe de problèmes de dirichlet non linéaires. Revue française d'automatique, informatique, recherche opérationnelle. Analyse numérique **9**(2), 41–76 (1975)
8. Nievergelt, Y.: Hyperspheres and hyperplanes fitted seamlessly by algebraic constrained total least-squares. Linear Algebra and its Applications **331**(1), 43–59 (2001)
9. Liang, J., Fadili, J., Peyré, G.: Convergence rates with inexact non-expansive operators. Mathematical Programming **159**(1-2), 403–434 (2016)
10. Fitzgibbon, A., Pilu, M., Fisher, R.B.: Direct least square fitting of ellipses. IEEE Transactions on pattern analysis and machine intelligence **21**(5), 476–480 (1999)

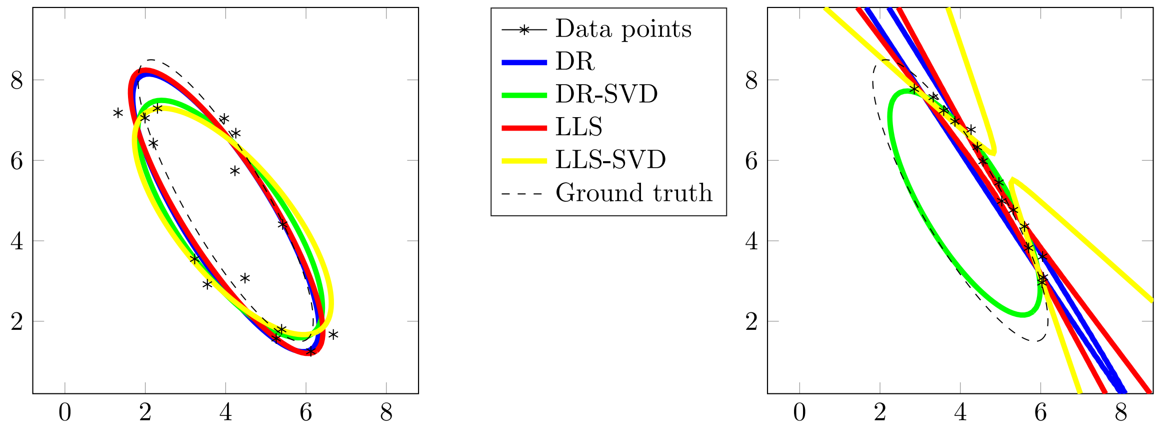

Figure 3 Comparisons of 4 different algorithms to fit an ellipse to a set of points. Left: all algorithms perform well for uniformly distributed points along an ellipse. The number of iterations necessary for DR to converge is 4000 and it is 30 for DR-SVD. Right: results for nonuniformly distributed points. The LLS and LLS-SVD algorithm produce hyperbolas instead of ellipses. In this specific case, DR-SVD produces a result closer to the ground truth than DR, but it is not the case for all noise realizations. Notice that the users of our plugins should avoid specifying configurations of points as shown in the right figure.

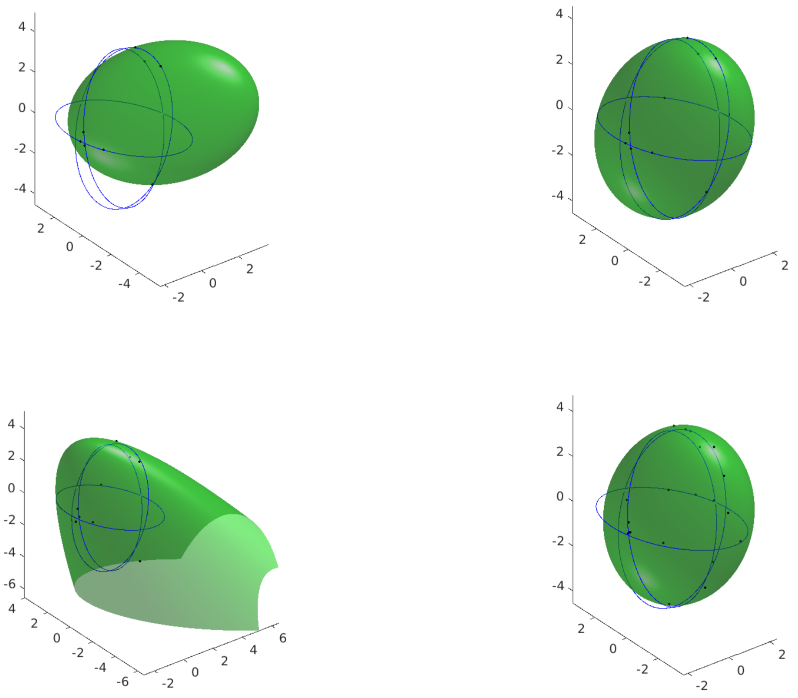

Figure 4 Fitting a 3D ellipsoid with semi-axes lengths $l_1 = 5$, $l_2 = 3$ and $l_3 = 1$. All results in this experiment were obtained with the proposed DR-SVD algorithm. The retrieved ellipsoid is displayed in green. The planar sections of the true underlying ellipsoid are displayed in blue. The sampled points are displayed in black. In each experiment, numerical precision was obtained in less than 200 iterations. Top-left ($n = 8$ points, $\sigma = 0$) : this is not sufficient to retrieve the ellipsoid. Top-right ($n = 9$, $\sigma = 0$): this is sufficient to perfectly recover the exact ellipsoid. These two results illustrate Proposition 1. Bottom-left ($n = 9$, $\sigma = 0.2$): the detection is inaccurate since n is too small. Bottom-right ($n = 20$, $\sigma = 0.2$): increasing the number of points n renders satisfactory results even in noisy settings.
